# Mixing Mechanism of Microfluidic Mixer with Staggered Virtual Electrode Based on Light-Actuated AC Electroosmosis

**DOI:** 10.3390/mi12070744

**Published:** 2021-06-24

**Authors:** Liuyong Shi, Hanghang Ding, Xiangtao Zhong, Binfeng Yin, Zhenyu Liu, Teng Zhou

**Affiliations:** 1 Mechanical and Electrical Engineering College, Hainan University, Haikou 570228, China; shiliuyong@hainanu.edu.cn (L.S.); dinghanghang@hainanu.edu.cn (H.D.); zhongxiangtao97@foxmail.com (X.Z.); 2 School of Mechanical Engineering, Yangzhou University, Yangzhou 225127, China; binfengyin@yzu.edu.cn; 3 Changchun Institute of Optics, Fine Mechanics and Physics (CIOMP), Chinese Academy of Sciences, Changchun 130033, China; liuzy@ciomp.ac.cn

**Keywords:** light-actuated AC electroosmosis (LACE), microfluidic mixer, optical virtual electrode, electrokinetics, computational fluid dynamics (CFD)

## Abstract

In this paper, we present a novel microfluidic mixer with staggered virtual electrode based on light-actuated AC electroosmosis (LACE). We solve the coupled system of the flow field described by Navier–Stokes equations, the described electric field by a Laplace equation, and the concentration field described by a convection–diffusion equation via a finite-element method (FEM). Moreover, we study the distribution of the flow, electric, and concentration fields in the microchannel, and reveal the generating mechanism of the rotating vortex on the cross-section of the microchannel and the mixing mechanism of the fluid sample. We also explore the influence of several key geometric parameters such as the length, width, and spacing of the virtual electrode, and the height of the microchannel on mixing performance; the relatively optimal mixer structure is thus obtained. The current micromixer provides a favorable fluid-mixing method based on an optical virtual electrode, and could promote the comprehensive integration of functions in modern microfluidic-analysis systems.

## 1. Introduction

Micromixing [1] is one of the key technologies of micro-total analysis systems (μTAS) [2] and lab-on-a-chip (LOC) [3] devices, and it is widely used in the fields of biology [4], chemistry [5], medicine [6], and engineering, both in academia and industry, because of its low cost, high speed, and low sample consumption [7,8,9]. However, compared with macrofluids, microfluidic flow is highly ordered laminar flow due to the low Reynolds numbers, and molecular diffusion is the main mechanism of microfluidic mixing [10]. Small molecules (rapidly diffusing species) can achieve diffusion mixing within tens of microns during a few seconds, but the equilibrium time required for mixing large molecules (peptides, proteins, and high-molecular-weight nucleic acids) within the same distance is from several minutes to several hours. For many chemical analyses, this means that longer channel lengths and mixing times are required to achieve the homogenization of the species concentration, and such delays are impractical. Therefore, to improve the mixing efficiency of the micromixer by optimizing the size of the device and the time of sample analysis is the main object of the design and development of micromixers today.

According to mixing mechanism, micromixers can be basically divided into active [11] and passive [12,13] micromixers. Passive micromixers rely on the geometry of microchannels to generate complex flow fields, such as a flow split [14], flow recombination [15], flow separation, chaotic advection [16], and hydrodynamic focus to realize the efficient mixing of fluids [17,18]. Active micromixers, via external energy or stimuli, improve the mixing of fluids either with moving parts [19] or with external force fields such as acoustic [20,21], electric [22,23,24], magnetic [25,26], thermal [27,28,29,30], and pressure [31] fields. The rotating electroosmotic vortex generated under the action of applied potential is widely used in active micromixers to manipulate and control different fluids to achieve mixing. In the past few years, a large number of electroosmotic microfluidic mixers were designed and developed [32]. In general, when a fixed microelectrode [33] is embedded on the sidewall of the microchannel, a pair of electroosmotic rotating vortices can be generated near the microelectrode to achieve the mixing of fluids in the microchannel [34]. The implantation of a heterogeneous surface charge [35,36,37,38] and a nonuniform surface potential charge [39] on the side and/or bottom walls of the microchannel to induce localized nonaxial flow structure or vortices can achieve better mixing efficiency [40]. However, the method of embedding fixed microelectrodes or performing heterogeneous processing on the inner wall of the microchannel both increases the difficulty and cost of processing the microchannel, and reduces the flexibility of microchip functionality.

With the development of light-induced technology [41,42,43], a rotating vortex generated by LACE can both manipulate microparticles in the fluids [44,45] and be used for the mixing of different fluids [46]. However, the mixer structure and mixing efficiency in [46] could still be further optimized and improved. On this basis, we improved the inlet layout of the mixer and designed a new type of staggered electrode, further revealing the fluid-mixing mechanism under the combined action of the staggered electrode and the applied electric field by multiphysics field coupling numerical simulation. Moreover, the influence of geometric parameters, including the length, width, and spacing of the staggered virtual electrodes, and the height of the microchannel, on mixing efficiency was studied (this is rarely mentioned in previous studies, and the circular spot or ring virtual electrode was generally employed before); thus, a relatively optimal mixer structure was obtained.

## 2. Theory and Methods

### 2.1. Micromixer Structure

**Figure 1:**
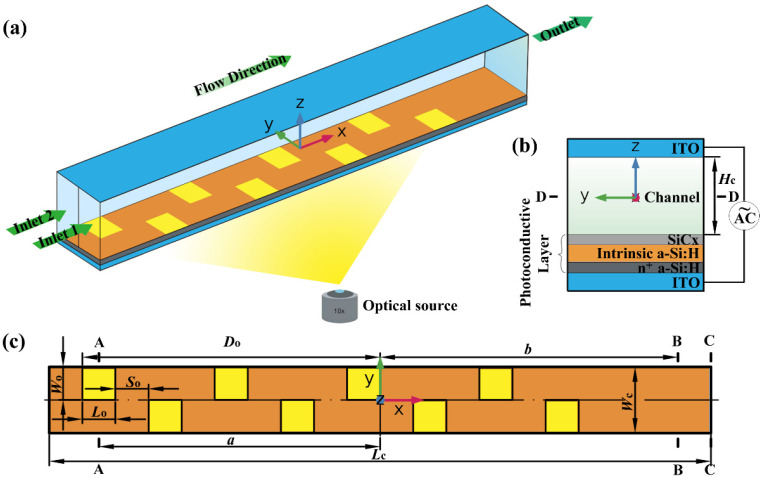
Microfluidic mixer with staggered virtual electrode based on LACE. (**a**) Three-dimensional structure of micromixer; green arrows indicate flow direction. (**b**) Cross-sectional (yz section) view of micromixer and photoconductive layer. (**c**) Top view of micromixer. Orange and yellow areas indicate photoconductive layer and the staggered light spots projected onto photoconductive layer, respectively. A–A, B–B, and C–C represent cross-section (yz section) at a distance of −85, 90, and 100 μm (exit position) from microchannel center, respectively. D–D represents section (xy section) at the microchannel center. Origin of the Cartesian coordinate system is located at the microchannel center.

Figure 1a shows the schematic diagram of a three-dimensional structure of the microfluidic mixer with staggered virtual electrode based on LACE. In this model, two kinds of fluids with the same solute but different solute concentrations (*c*_1_ = 0 mol/m^3^, *c*_2_ = 1 mol/m^3^) were driven by pressure from Inlets 1 and 2 into the straight mixing microchannel. The length of the straight microchannel was *L*_c_ = 200 μm, and width and height were *W*_c_ = *H*_c_ = 20 μm. The center line on the inlet boundary perpendicular to the bottom wall of the microchannel was taken as the dividing line between Inlets 1 and 2. As shown in Figure 1b, the top and bottom walls of the microchannel were transparent indium tin oxide (ITO) glass. The photoconductive layer was coated at the surface of the bottom wall of the microchannel by plasma-enhanced chemical vapor deposition (PECVD). From top to bottom, the photoconductive layer was a multilayer film structure composed of SiC_x_ film with a thickness of 25 nm, intrinsic a-Si:H with a thickness of 2 μm, and n^+^ a-Si:H with a thickness of 50 nm [47].

In general, the conductivity of the photoconductive layer was approximately *σ*_D_ = 6.7 × 10^–5^ S/m, and when light was projected onto the photoconductive layer, the conductivity of the illuminated area increased sharply to *σ*_L_ = 0.2 S/m [48]. This means that a specific nonuniform electric field was generated in the microchannel when a specific optical pattern was projected onto the photoconductive layer. Since this optical pattern could produce a nonuniform electric field similar to the fixed metal microelectrode, it could called the “optical virtual electrode”. The generation and adjustment of the virtual electrode could be realized by a series of optical lenses and plane mirrors based on a digital micromirror device (DMD). The position of the virtual electrodes staggered along the axial direction of the microchannel is shown in Figure 1c, which clearly shows the characteristic size (*L*_c_, *W*_c_) of the microchannel, the characteristic size (*L*_o_ = 10 μm, *W*_o_ = 10 μm, *S*_o_ = 10 μm, *D*_o_ = 90 μm) of the staggered light spot projected onto the photoconductive layer, and the position of each section (A–A, B–B, C–C, D–D). In this study, the number of staggered virtual electrodes is 8.

### 2.2. Governing Equations

The transient Navier–Stokes equations for incompressible fluids describe the flow in the microchannel:

(1) $$\rho \frac{\partial u}{\partial t}-\nabla \cdot \mu \left(\nabla u+{\left(\nabla u\right)}^{T}\right)+\rho u\cdot \nabla u+\nabla p=0$$

(2) ∇⋅u=0

where *ρ* is density, *μ* is dynamic viscosity, ***u*** is velocity, *σ* is conductivity, and *p* is pressure. *ρ* = 1000 kg/m^3^, *μ* = 0.001 kg/(m∙s), and *σ* = 0.11845 S/m when we use an aqueous solution as fluid medium.

Uniform fluid velocity was applied at inlet boundaries 1 and 2:

(3) $$u_{1}=U_{1}n$$

(4) $$u_{2}=U_{2}n$$

Here, *U*_1_ and *U*_2_ are the fluid velocities at Inlets 1 and 2, respectively; ***n*** is the normal vector of the inlet boundary; *U*_m_ = *U*_1_ = *U*_2_ = 2 × 10^–4^ m/s is the mean inlet velocity.

The mixed fluid freely flowed out from the outlet boundary of the microchannel. The total stress component was perpendicular to boundary

(5) $$\left(-pI+\mu \left(\nabla u+{\left(\nabla u\right)}^{T}\right)\right)\cdot n=0,$$

(6) p=0

where ***I*** is the unit tensor.

Compared with the characteristic size of the microchannel, the thickness (Debye length) of the electrical double layer on the inner wall surface of the microchannel was very small, so the Helmholtz–Smoluchowski slip-boundary condition between electroosmotic velocity and the tangential component of the applied potential could be applied to the entire top and bottom walls of the microchannel:

(7) $$u_{\mathrm{slip}}=-\frac{\varepsilon_{0}\varepsilon_{f}\zeta }{\mu }E_{t}$$

where *ζ* = −0.1 V is the zeta potential on the top and bottom walls of the microchannel; ***E***_t_ is the tangential component of the applied potential vector; *ε*_0_ is the vacuum dielectric constant; and *ε_f_* = 80.2 is the relative permittivity of the fluid.

Symmetrical boundary conditions applied to the two side walls of the microchannel described no penetration and vanishing shear stresses.

Assuming that there was no concentration gradient in the ions carrying current, a Laplace equation was used to describe the applied potential:

(8) $$\nabla^{2}\varphi =0$$

(9) E=−∇φ

***E*** is the applied electric-field intensity vector, and *φ* is the applied potential.

(10) $$\varphi =V_{0}\sin\left(2\pi ft\right)$$

*V*_0_ = 1 V is the peak-to-peak value of the applied potential, and *f* = 5 Hz is the electric-field frequency. The duration of one electric-field cycle was *T* = 1/*f* = 0.2 s. The top wall of the microchannel was grounded.

All other boundaries were electrically insulated:

(11) n⋅∇φ=0

In the microchannel, the convection–diffusion equation is used to describe the concentration of dissolved species in the fluid:

(12) $$\frac{\partial c}{\partial t}+\nabla \cdot \left(-D\nabla c\right)=-u\cdot \nabla c$$

where *c* is the species concentration, and *D* = 10^–11^ m^2^/s [11] is the diffusion coefficient of the solute.

At Inlets 1 and 2, the solute gave concentrations of *c*_1_ = 0 mol/m^3^ and *c*_2_ = 1 mol/m^3^.

The mixed fluid flowed out from the outlet by convection, and the boundary conditions along the outlet were as follows:

(13) (D∇c)⋅n=0

All other boundaries were set to no flux:

(14) (cu−D∇c)⋅n=0

The mixing efficiency of the two fluids near the outlet of the microchannel was calculated by using the following formula [49]:

(15) $$M=1-\sqrt{\frac{{\iint_{\Gamma }\left(c-\bar{c}\right)}^{2}}{S{\bar{c}}^{2}}}$$

where *Γ* is the cross-section used to measure the concentration. To avoid the outlet effect, the B–B cross-section was selected here. *c* and $\bar{c}$ are the concentration and average concentration on *Γ,* respectively. *S* is the area of the *Γ*. *M* = 0 and *M* = 1 mean in which the two fluids were completely separated and mixed. Therefore, the greater the value of *M* was, the better the mixing effect of the two fluids was.

**Figure 2:**
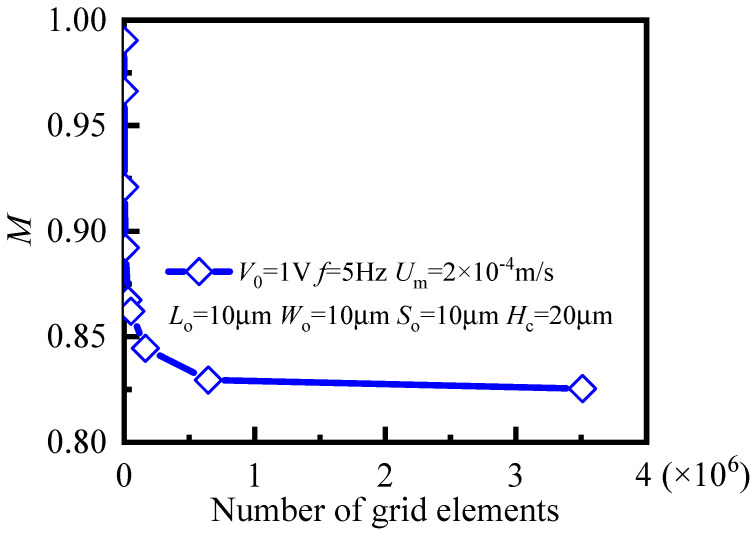
Grid-dependency test for mixing-efficiency index *M* at B-B cross-section.

In this paper, commercial finite-element software package COMSOL Multiphysics (Version 5.3a, COMSOL Group, Stockholm, Sweden) was used to solve the coupling system of laminar flow, electric currents, and transport of diluted species. To ensure the credibility of the simulation results, a grid-dependency test was carried out to determine the optimal number of grid elements. We calculated mixing-efficiency index *M* of the B-B cross-section when *t* = 4 T for 9 different numbers of grid elements from 750 to 3,510,190, and results are shown in Figure 2. The mixing-efficiency index remained unchanged when the number of grid elements exceeded 612,909. Therefore, considering the accuracy and efficiency of simulation, 612,909 was selected as the optimal number of grid system elements. According to the data statistics of COMSOL 5.3a, the numbers of hexahedral, quads, edge, and vertex elements for the required calculation in the simulation model were 612,909, 80,127, 3652, and 76, respectively. Maximal and minimal element sizes were 0.544 and 0.0355 μm, respectively. The minimal element quality and average element quality could reach 0.5766 and 0.9812, respectively. This proves that the solution and analysis of the simulation model in this grid system were relatively accurate and reliable.

## 3. Results and Discussion

### 3.1. Mixing Process and Mechanism

**Figure 3:**
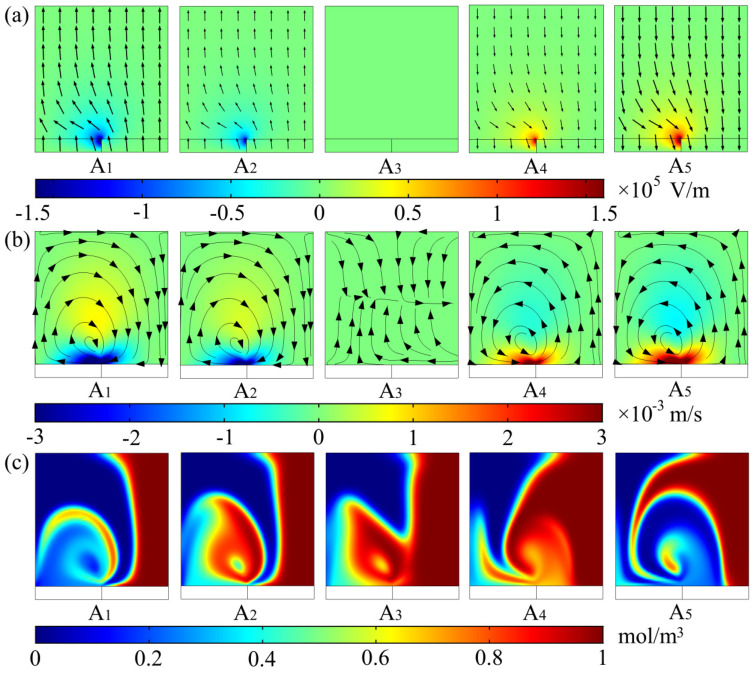
(**a**) *Y* component of applied potential, (**b**) flow field, and (**c**) concentration-distribution evolution of A–A cross-section in the microchannel at five different moments during one cycle. A, cross-section in the microchannel, and subscript i represents different moments (A_1_) *t* = 1/4 T, (A_2_) *t* = 3/8 T, (A_3_) *t* = 1/2 T, (A_4_) *t* = 5/8 T and (A_5_) *t* = 3/4 T. Peak-to-peak value of applied potential was *V*_0_ = 1 V, electric-field frequency was *f* = 5 Hz, and inlet mean velocity was *U*_m_ = 2 × 10^–4^ m/s. Length was *L*_o_ = 10 μm, width was *W*_o_ = 10 μm and spacing was *S*_o_ = 10 μm of the virtual electrode. Aspect ratio of the microchannel was *W*_c_/*H*_c_ = 1. (**a**) Black arrows, electric-field intensity vectors; and color legend, magnitude of *E*_y_. (**b**) Black lines, streamlines; black arrows, flow direction; color legend, magnitude of velocity component in *y* direction. (**c**) Color legend, concentration distribution from 0 to 1 mol/m^3^.

The famous Helmholtz–Smoluchowski slip-boundary condition (Equation (7)) shows that the magnitude of electroosmotic slip velocity *u*_slip_ is closely related to tangential component *E*_t_ of the applied potential. In addition, tangential component *E*_t_ of the total electric field on the top and bottom walls of the microchannel included *E*_x_ and *E*_y_, considering that the slip velocity resulting from *E*_x_ had little perturbance on the main flow direction, so the generation of electroosmotic flow on the cross-section mainly depended on *E*_y_. Thus, by giving the electric-field, flow-field, and concentration-distribution evolution (as shown in Figure 3) of the A–A cross-section in the microchannel at five different moments during one electric-field cycle, we elaborate the effect of the staggered virtual electrodes on the fluid mixing and reveal its mixing mechanism. Here, the selected A–A cross-section was located at the center of the virtual electrode adjacent to the inlet of the microchannel, as shown in Figure 1c. Since a sinusoidal AC signal was applied in this study, the five different taken moments were *t* = 1/4 T, *t* = 3/8 T, *t* = 1/2 T, *t* = 5/8 T and *t* = 3/4 T.

A_1_, A_2,_ and A_3_ in Figure 3a show that, at the middle moment of the first half cycle (*t* = 1/4 T), the *y* component of the electric field in the A–A section became the largest, and it was weakened when *t* = 3/8 T, until it completely decreased to 0 V/m at the end of the first half cycle (*t* = 1/2 T). A_4_ and A_5_ show that the magnitude of *E*_y_ gradually increased when *t* = 5/8 T, and reaches the maximal value again at the middle moment of the second half cycle (*t* = 3/4 T), which was basically equal to that of the first half cycle. Different from the first half cycle, the direction of *E*_y_ in the second half cycle was the opposite. Due to the different voltage drop between the illuminated and dark areas in the photoconductive layer, the electric field on the A–A section exhibited nonuniform distribution, and it was larger near the boundary between the illuminated and dark areas. In addition, since the virtual electrodes on the bottom wall of the microchannel were arranged in a staggered manner, electric-field distribution on the cross-sections of the two staggered electrodes adjacent to each other at the same time was basically equal in magnitude, and exactly the opposite in direction.

As shown in A_1_ in Figure 3b, when *t* = 1/4 T, a clockwise rotating vortex with an influence range of almost the entire microchannel was generated on the A–A section. The center of the rotating vortex was located near the boundary between the illuminated and dark areas with large flow velocity. A_2_ shows that the size and rotation direction of the rotating vortex were basically unchanged when *t* = 3/8 T, while the magnitude of the velocity near the bottom wall of the microchannel was smaller than that when *t* = 1/4 T. A_3_ shows that, when *t* = 1/2 T, the fluid in the microchannel returned to highly ordered laminar flow, that is, there was no electroosmotic flow generated on A–A section. A_4_ shows that the rotation direction of the rotating vortex was counterclockwise when *t* = 5/8 T, which is opposite to that of the first half cycle. When *t* = 3/4 T from A_5_, the magnitude of the velocity of the rotating vortex reached the maximum again, and the rotation direction was still opposite to that of the first half cycle. The reason for the above changes in the flow field is that the magnitude of the electroosmotic slip velocity was proportional to the tangential component of the applied electric-field intensity. From the perspective of the entire cycle, the size and direction of the rotating vortex generated in the microchannel were closely related to the magnitude and direction of *E*_y_, which is consistent with the description in Figure 3a.

As shown in A_1_ in Figure 3c, the rotating vortex generated on the A–A section strongly disturbed the interface between the two fluids when *t* = 1/4 T. The fluid elements were folded, and stretched widely and repeatedly in the direction perpendicular to the main flow, and the two fluids were wrapped around each other A_2_ and A_3_ show that the mixing area of the two fluids was still expanding from *t* = 3/8 T to *t* = 1/2 T, although the rotating vortex gradually decayed until it disappears. A_4_ and A_5_ show that the fluid elements were folded and stretched in opposite directions due to the change in the rotational direction of the rotating vortex; when *t* = 5/8 T, the two fluids were wrapped layer by layer until *t* = 3/4 T. On the whole, the alternating rotating vortex on A–A section strengthened the convection between the two fluids and increased the turbulence of fluid motion, but the two fluids on A–A section were still well-separated. This shows that only one virtual electrode could not achieve a high level of mixing performance in the whole microchannel.

**Figure 4:**
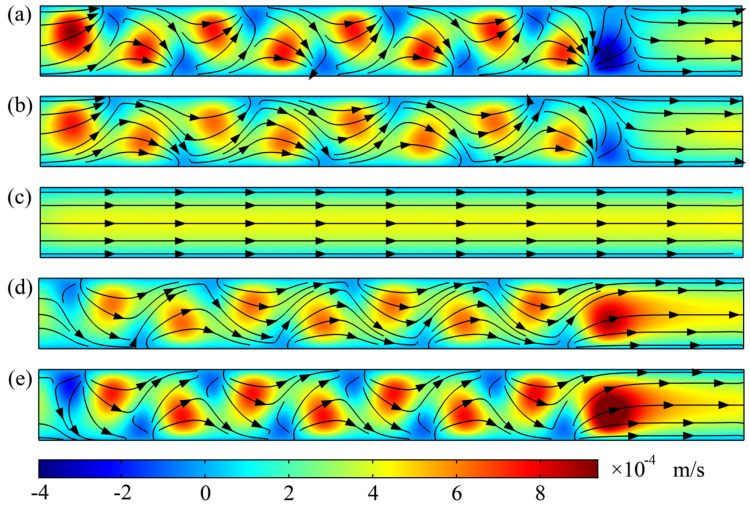
Flow fields of D–D section in the microchannel at five different moments during one cycle: (**a**) *t* = 1/4 T, (**b**) *t* = 3/8 T, (**c**) *t* = 1/2 T, (**d**) *t* = 5/8 T and (**e**) *t* = 3/4 T. Peak-to-peak value of applied potential was *V*_0_ = 1 V, electric-field frequency was *f* = 5 Hz, and inlet mean velocity was *U*_m_ = 2 × 10^–4^ m/s. Length was *L*_o_ = 10 μm, width was *W*_o_ = 10 μm, and spacing was *S*_o_ = 10 μm of the virtual electrode. Aspect ratio of the microchannel was *W*_c_/*H*_c_ = 1. Black lines, streamlines; black arrows, flow direction; color legend, magnitude of the velocity component in the *x* direction.

To explore the effect of the rotating vortex generated on the cross-section on the axial mainstream of the microchannel and further reveal its mixing mechanism, we studied the flow fields of the D–D section in the microchannel at five different moments during one cycle (as shown in Figure 4). Here, the selected five different moments were consistent with those in Figure 3. As shown in Figure 1b, the D–D section was the *xy* section when *z* = 0.

Figure 4a shows that, under the action of the rotating vortex generated on the cross-section of the microchannel, the direction of the main flow was significantly deflected when *t* = 1/4 T, and the maximal deflection angle was almost close to 90 degrees. In addition, due to the staggered arrangement of the virtual electrodes on the bottom wall of the microchannel, the main flow deflected to the opposite direction every time it passed through a virtual electrode, which greatly enhanced the convection between two fluids. Since there was no spot irradiation near the outlet of the microchannel, the fluid near the outlet returned to a highly ordered laminar flow. Figure 4b shows that the main flow still deflected when passing through the electrode (*t* = 3/8 T), but the degree of deflection became smaller, which is related to the decrease in flow velocity of the rotating vortex generated on the cross-section. The flow field when *t* = 1/2 T in Figure 4c shows that the main flow in the whole microchannel was restored to highly orderly laminar flow since the electric field was zero at this time. The effect of fluid-relaxation time was not considered here because of the low electric-field frequency. Figure 4d shows that, under the action of the rotating vortex with opposite rotation direction compared with that in the first half cycle, the main flow direction deflected again when *t* = 5/8 T, and the deflection direction of the fluid at the same position was opposite to that of the first half cycle. As shown in Figure 4e, the deflection angle of the mainstream again reached the maximum when *t* = 3/4 T. Meanwhile, under the pumping action of *E*_x_, the fluid near the outlet could quickly pass through the outlet of the microchannel.

Under the coupling action of an alternating electric field and a series of staggered virtual electrodes, the mainstream both flowed along the S-shaped path in the microchannel, and also periodically and alternately changed with time, which greatly improved the convection between the two fluids and positively affected fluid mixing.

**Figure 5:**
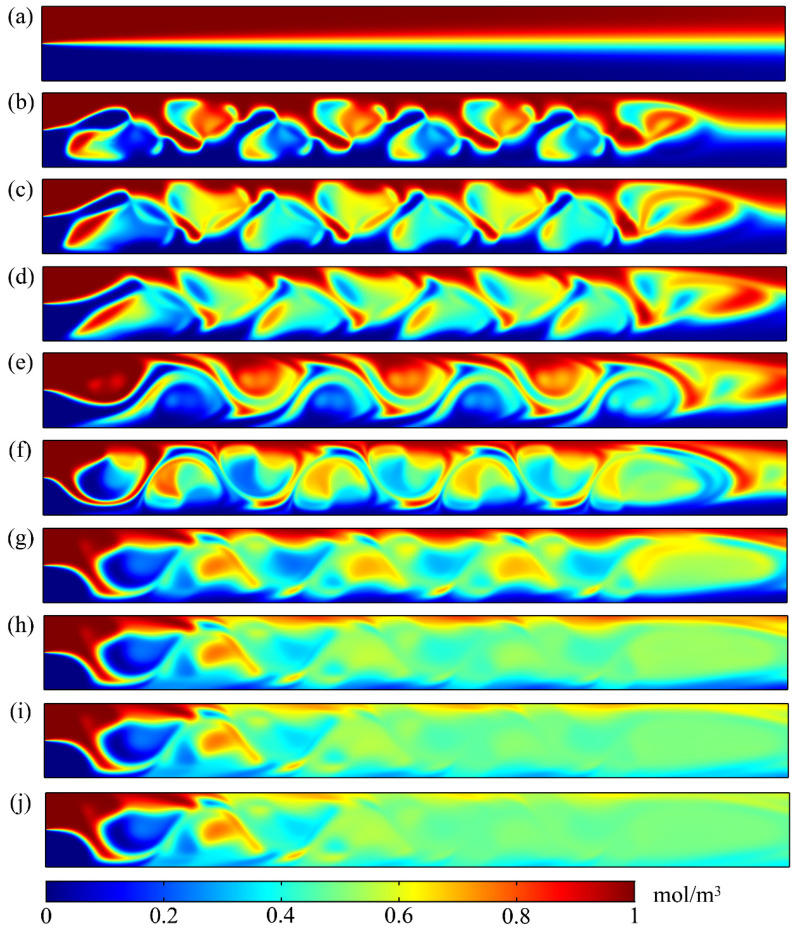
Concentration-distribution evolution of D–D section in microchannel at 10 different moments during 4 cycles: (**a**) *t* = 0 T, (**b**) *t* = 1/4 T, (**c**) *t* = 3/8 T, (**d**) *t* = 1/2 T, (**e**) *t* = 5/8 T, (**f**) *t* = 3/4 T, (**g**) *t* = 1 T, (**h**) *t* = 2 T, (**i**) *t* = 3 T, and (**j**) *t* = 4 T. Peak-to-peak value of applied potential was *V*_0_ = 1 V, electric-field frequency was *f* = 5 Hz, and inlet mean velocity was *U*_m_ = 2 × 10^–4^ m/s. Length was *L*_o_ = 10 μm, width was *W*_o_ = 10 μm, and spacing was *S*_o_ = 10 μm of the virtual electrode. Aspect ratio of the microchannel was *W*_c_/*H*_c_ = 1. Color legend, concentration distribution from 0 to 1 mol/m^3^.

Figure 5 shows the concentration-distribution evolution of the D–D section in the microchannel at 10 different moments during four cycles. The 10 different selected moments were (a) *t* = 0 T, (b) *t* = 1/4 T, (c) *t* = 3/8 T, (d) *t* = 1/2 T, (e) *t* = 5/8 T, (f) *t* = 3/4 T, (g) *t* = 1 T, (h) *t* = 2 T, (i) *t* = 3 T, and (j) *t* = 4 T.

Figure 5a shows that, at the initial moment (*t* = 0 T), molecular diffusion at the interface between the two fluids in the microchannel was the main mixing mechanism, so the two fluids were well-separated. As shown in Figure 5b, in the vicinity of the virtual electrode, the rotating vortex generated on the cross-section disturbed the interface between the two fluids when *t* = 1/4 T, which made the fluid elements in the D–D section fold, and widely and repeatedly stretch in the direction perpendicular to the mainstream, and the two fluids were wrapped around each other. Figure 5c shows that, although the velocity of the rotating vortex on the cross-section decreased due to the attenuation of the electric field when t = 3/8 T, the interface between the two fluids was serrated. The convection between the fluids was gradually strengthened, and the mixing area dramatically expanded. Figure 5d shows that the rotating vortex on the cross-section disappeared when *t* = 1/2 T, but the mixing area was still expanding under the action of the mainstream. Figure 5e shows that, when *t* = 5/8 T, due to the opposite rotation vortex in the cross-section compared with that in the first half cycle, the main flow stretched and folded in the opposite direction, and the interface between the two fluids gradually presented an arc shape. Figure 5f shows that, under the action of the gradually increasing applied potential, the length of the fluid interface increased when *t* = 3/4 T, and the two fluids in the microchannel were wrapped around each other.

Although the two fluids on the D–D section were alternately folded and stretched under the action of the rotating vortex on the cross-section, the two fluids still had a clear dividing line during one cycle, as shown in Figure 5g. Figure 5h shows that, when *t* = 2 T, the fluids were basically mixed except those near the inlet and side wall of the microchannel. When *t* = 3 T, as shown in Figure 5i, the fluids near the side wall of the microchannel gradually realized mixing. Figure 5j shows that, when *t* = 4 T, the fluids after passing through the virtual electrode were basically completely mixed. Figure 5 shows that the mixing effect of the fluids gradually improved as the applied potential was prolonged. In addition, the two fluids near the inlet are always well separated. As the number of virtual electrodes through which the fluids flowed increased, the mixing effect of the fluids became more favorable, and the two fluids basically achieved complete mixing near the outlet.

### 3.2. Influence of Key Geometric Parameters on Mixing Performance

In this section, we evaluate the mixing performance of the micromixer by studying the concentration-distribution evolution near the outlet of the microchannel. To avoid the outlet effect, we chose the B–B section within the microchannel, which was 10 μm away from the outlet, as shown in Figure 1c.

**Figure 6:**
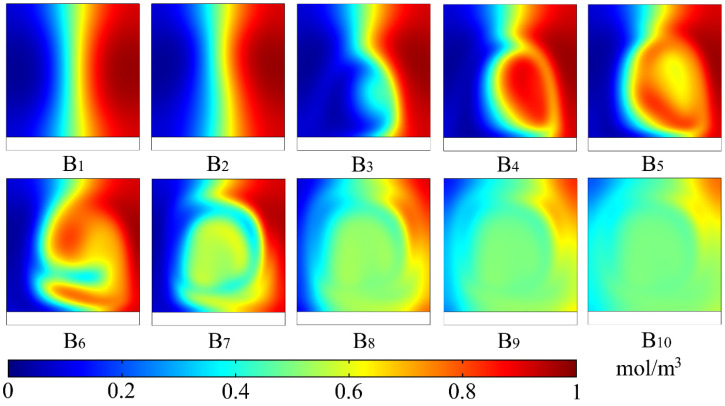
Concentration-distribution evolution of B–B cross-section (near the outlet) at 10 different moments in the microchannel during four cycles. B, cross-section; subscript i, different moments: (**B_1_**) *t* = 0 T, (**B_2_**) *t* = 1/4 T, (**B_3_**) *t* = 3/8 T, (**B_4_**) *t* = 1/2 T, (**B_5_**) *t* = 5/8 T, (**B_6_**) *t* = 3/4 T, (**B_7_**) *t* = 1 T, (**B_8_**) *t* = 2 T, (**B_9_**) *t* = 3 T and (**B_10_**) *t* = 4 T. Peak-to-peak value of applied potential was *V*_0_ = 1 V, electric-field frequency was *f* = 5 Hz, and inlet mean velocity was *U*_m_ = 2 × 10^–4^ m/s. Length was *L*_o_ = 10 μm, width was *W*_o_ = 10 μm, and spacing was *S*_o_ = 10 μm of the virtual electrode. Aspect ratio of the microchannel was *W*_c_/*H*_c_ = 1. Color legend, concentration distribution from 0 to 1 mol/m^3^.

The concentration-distribution evolution of the B–B cross-section in the microchannel at ten different moments during four cycles is shown in Figure 6. Here, the 10 different selected moments were consistent with those in Figure 5. As shown in B_1_, the two fluids near the outlet presented highly ordered laminar flow at the initial moment (*t* = 0 T). B_2_ indicated that, although electric-field intensity in the microchannel reached the maximum when *t* = 1/4 T, the two fluids were still well-separated because of the short duration of the rotating vortex in the initial stage. B_3_ shows that, when *t* = 3/8 T, although the electric field gradually weakened, slight distortion occurred at the interface between the two fluids under the action of the continuous rotating vortex. B_4_ indicates that there was no electric field in the microchannel when *t* = 1/2 T, but the interface between the two fluids was significantly distorted. B_5_ shows that the direction of all rotating vortices in the cross-section of the microchannel changed when *t* = 5/8 T, and the interface between the two fluids was distorted in opposite directions. B_6_ shows that, under the action of continuously changing applied potential, with the increase in electric-field intensity in the microchannel, the two fluids could wrap around each other when *t* = 3/4 T. B_7_ shows that, under the action of applied potential during one cycle (*t* = 1 T), the two fluids realized mutual wrapping. B_8_ indicates that, with the periodic change in applied potential, except those near the side wall of the microchannel, the two fluids achieved a better mixing effect at the end of the second cycle (*t* = 2 T). B_9_ and B_10_ show that the mixing degree of the two fluids in the microchannel became increasingly uniform by increasing the duration time of the applied potential, and the two fluids were basically completely mixed at the end of the fourth cycle (*t* = 4 T).

**Figure 7:**
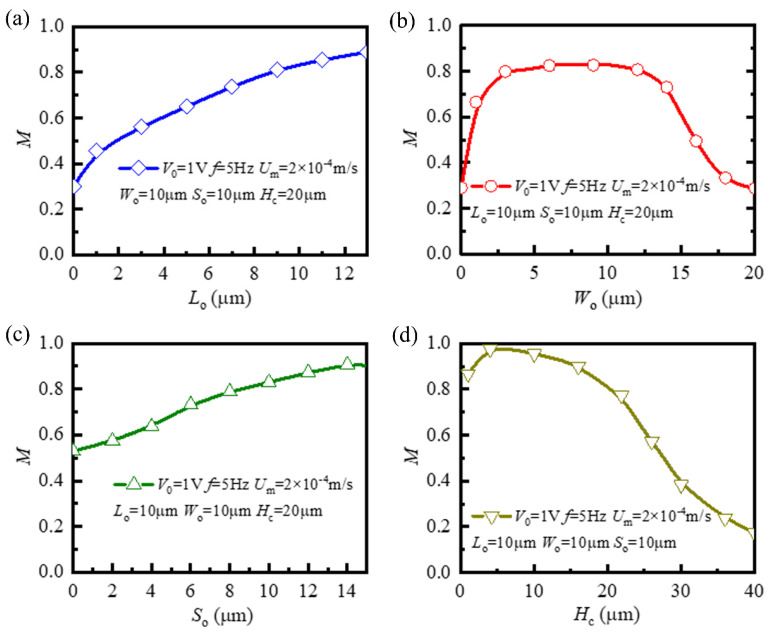
Mixing-efficiency index *M* near outlet depending on different geometric parameters. (**a**) Length *L*_o_, (**b**) width *W*_o_, (**c**) spacing *S*_o_ of virtual electrode; (**d**) height *H*_c_ of the microchannel.

To obtain the optimal mixing performance, we continued to study the influence of several key geometric parameters on mixing performance, namely, length *L*_o_, width *W*_o_, and spacing *S*_o_ of the virtual electrode, and height *H*_c_ of the microchannel. Here, we apply mixing-efficiency index *M* (Equation (15)) calculated on the B–B section at the end of the fourth cycle (*t* = 4 T) as the evaluation criterion of mixing performance. The dependence of mixing-efficiency index *M* on length *L*_o_ of the virtual electrode is shown in Figure 7a. Mixing-efficiency index *M* increased with the increase in length *L*_o_ of the virtual electrode. When the length of the virtual electrode was *L*_o_ = 0 μm (no virtual electrode), due to the absence of an electroosmotic rotating vortex, the fluid hardly had any additional mixing effect (*M* = 0.290). However, with the increase in the length of the virtual electrode, the axial length of the electroosmotic rotating vortex generated on the cross-section of the microchannel became larger. The rotating vortex with larger axial length had stronger stability and greater distortion to the main flow in the microchannel. For instance, when *L*_o_ was equal to 13 μm, the efficiency reached the maximal value (*M* = 0.889).

Figure 7b shows the variation of mixing-efficiency index *M* with width *W*_o_ of the virtual electrode. As the width of the virtual electrode increased, mixing-efficiency index *M* generally first increased, reached the maximum when width *W*_o_ was about half of the width of the channel, and then decreased. According to the study of the electric field on the cross-section of the microchannel and the resulting electroosmotic rotating vortex in Figure 3, the center of the rotating vortex was located approximately at the boundary between the illuminated and dark areas. This means that only when the center of the rotating vortex was close to the center of the microchannel, was the disturbance degree to the fluid interface the maximum, and this achieved the best mixing effect. When width *W*_o_ was excessively large or small, the center of the rotating vortex was located inside the fluid on one side, which was not conducive to mixing the two fluid samples.

Figure 7c shows variation in mixing-efficiency index *M* with spacing *S*_o_ of the virtual electrode. As the distance between two adjacent virtual electrodes increased, the mixing-efficiency index basically linearly also increased. Figure 3 shows that the rotating vortex was generated on the cross-section where each virtual electrode was located at the microchannel. At the same time, the rotation direction of the rotating vortex on the adjacent cross-section was the opposite because the virtual electrodes were staggered in this study. When the fluids in the mainstream direction flowed through two adjacent virtual electrodes in turn, under the action of two rotating vortices with opposite rotating directions perpendicular to the mainstream direction, the interface between the two fluids at the two adjacent electrodes was distorted in opposite directions (as shown in Figure 4). However, when the two adjacent cross-sections were very close, such as *S*_o_ = 0 μm, the overall distortion of the two fluids in the main flow direction in the microchannel was weakened, which was not conducive to the mixing of the two fluids. This is consistent with the minimal value (*M* = 0.529) of the calculation results of the mixing-efficiency index in Figure 7c. With the increase in the space between the virtual electrodes, the overall distortion degree of the two fluids in the mainstream direction in the microchannel gradually increased. For example, when *S*_o_ = 15 μm, the two fluids in the mainstream direction were mixed to the greatest extent, and the mixing-efficiency index reached the maximal value (*M* = 0.904).

Mixing-efficiency index *M* changing with height *H*_c_ of the microchannel is shown in Figure 7d. Mixing-efficiency index *M* decreased with the increase in microchannel height. Figure 3 shows that the center of the rotating vortex on the cross-section of the microchannel was closer to the bottom wall of the microchannel, and the higher flow velocity was also concentrated near the boundary between the illuminated and dark areas. In addition, when the applied potential remained unchanged, the larger channel height resulted in a weaker electric field in the microchannel, which reduced electroosmotic slip velocity. Therefore, when the microchannel height increased, the disturbance of the rotating vortex to the interface between two fluids gradually weakened, which led to the deterioration of mixing performance. For example, when *H*_c_ = 40 μm, there was little mixing effect between the two fluids (*M* = 0.173). When the height of the microchannel was excessively small compared with its width (*H*_c_ < 5 μm, *W*_c_ = 20 μm), although electric-field intensity in the microchannel was large enough, the circular rotating vortex in the cross-section may have been compressed, resulting in a poor mixing effect of the fluids. Only when the height of microchannel was appropriately comparable with its width, such as *H*_c_ = 6 μm, could the rotating vortex that formed on the cross-section of the microchannel disturb the interface between the two fluids to the maximal extent, achieving the best mixing effect (*M* = 0.978).

## 4. Conclusions

In this paper, we presented a novel microfluidic mixer with staggered virtual electrode based on light-actuated AC electroosmosis. The coupling systems of Navier–Stokes equations, a Laplace equation, and a convection–diffusion equation were solved by a finite-element method. The flow field, electric field, and concentration distribution on the section of microchannel were studied. Simulation results showed that the electroosmotic rotating vortex generated on the cross-section of the microchannel enhanced convection and improved the mixing effect between fluids. In addition, we studied the effect of several key geometric parameters such as the length, width, and spacing of the staggered virtual electrode, and the height of the microchannel on mixing performance; the relatively optimal mixer structure was thus obtained (*L*_o_ = 13 μm, *W*_o_ = 10 μm, *S*_o_ = 15 μm, *H*_c_ = 6 μm). On the basis of its functional flexibility of optical virtual electrodes, the current fluid micromixer could promote the comprehensive integration of functions in modern microfluidic analysis systems. For example, reagent mixing, reaction, and detection steps may be concentrated in a single microfluidic channel.

## References

[1] Jeong G.S., Chung S., Kim C.B., Lee S.H. (2010). Applications of micromixing technology. Analyst.

[2] Manz A., Graber N., Widmer H.M. (1990). Miniaturized total chemical analysis systems a novel concept for chemical sensing. Sens. Actuators B1.

[3] Harrison D.J., Manz A., Fan Z., Luedi H., Widmer H.M., Manz J.A., Fan Z., Ludi H., Widmer H.M. (1992). Capillary electrophoresis and sample injection systems integrated on a planar glass chip. Anal. Chem..

[4] Alam M.K., Koomson E., Zou H., Yi C., Li C.W., Xu T., Yang M. (2018). Recent advances in microfluidic technology for manipulation and analysis of biological cells (2007–2017). Anal. Chim. Acta.

[5] Sasaki N., Kitamori T., Kim H.B. (2006). AC electroosmotic micromixer for chemical processing in a microchannel. Lab Chip.

[6] Jung J.H., Kim G.Y., Seo T.S. (2011). An integrated passive micromixer-magnetic separation-capillary electrophoresis microdevice for rapid and multiplex pathogen detection at the single-cell level. Lab Chip.

[7] Lee C.Y., Chang C.L., Wang Y.N., Fu L.M. (2011). Microfluidic mixing: A review. Int. J. Mol. Sci..

[8] Haeberle S., Zengerle R. (2007). Microfluidic platforms for lab-on-a-chip applications. Lab Chip.

[9] Mark D., Haeberle S., Roth G., von Stetten F., Zengerle R. (2010). Microfluidic lab-on-a-chip platforms: Requirements, characteristics and applications. Chem. Soc. Rev..

[10] Wang Y., Lin Q., Mukherjee T. (2005). A model for laminar diffusion-based complex electrokinetic passive micromixers. Lab Chip.

[11] Zhou T., Wang H., Shi L., Liu Z., Joo S.W. (2016). An enhanced electroosmotic micromixer with an efficient asymmetric lateral structure. Micromachines.

[12] Bayareh M., Ashani M.N., Usefian A. (2020). Active and passive micromixers: A comprehensive review. Chem. Eng. Process.-Process Intensif..

[13] Zhou T., Xu Y., Liu Z., Joo S.W. (2015). An enhanced one-layer passive microfluidic mixer with an optimized lateral structure with the dean effect. J. Fluids Eng..

[14] Hossain S., Kim K.-Y. (2015). Mixing analysis in a three-dimensional serpentine split and recombine micromixer. Chem. Eng. Res. Des..

[15] Sheu T.S., Chen S.J., Chen J.J. (2012). Mixing of a split and recombine micromixer with tapered curved microchannels. Chem. Eng. Sci..

[16] Chew Y.T., Xia H.M., Shu C., Wan S.Y.M. (2005). Techniques to enhance fluid micro-mixing and chaotic micromixers. Mod. Phys. Lett. B.

[17] Wang L., Liu D., Wang X., Han X. (2012). Mixing enhancement of novel passive microfluidic mixers with cylindrical grooves. Chem. Eng. Sci..

[18] Liu A.L., He F.Y., Wang K., Zhou T., Lu Y., Xia X.H. (2005). Rapid method for design and fabrication of passive micromixers in microfluidic devices using a direct-printing process. Lab Chip.

[19] Kim Y., Lee J., Kwon S. (2009). A novel micro-mixer with a quasi-active rotor: Fabrication and design improvement. J. Micromech. Microeng..

[20] Ahmed D., Mao X., Shi J., Juluri B.K., Huang T.J. (2009). A millisecond micromixer via single-bubble-based acoustic streaming. Lab Chip.

[21] Chen H., Chen C., Bai S., Gao Y., Metcalfe G., Cheng W., Zhu Y. (2018). Multiplexed detection of cancer biomarkers using a microfluidic platform integrating single bead trapping and acoustic mixing techniques. Nanoscale.

[22] Cartier C.A., Drews A.M., Bishop K.J. (2014). Microfluidic mixing of nonpolar liquids by contact charge electrophoresis. Lab Chip.

[23] Harnett C.K., Templeton J., Dunphy-Guzman K.A., Senousy Y.M., Kanouff M.P. (2008). Model based design of a microfluidic mixer driven by induced charge electroosmosis. Lab Chip.

[24] Wu Y., Ren Y., Tao Y., Hou L., Hu Q., Jiang H. (2016). A novel micromixer based on the alternating current-flow field effect transistor. Lab Chip.

[25] Yap L.W., Chen H., Gao Y., Petkovic K., Liang Y., Si K.J., Wang H., Tang Z., Zhu Y., Cheng W. (2017). Bifunctional plasmonic-magnetic particles for an enhanced microfluidic sers immunoassay. Nanoscale.

[26] Wen C.Y., Liang K.P., Chen H., Fu L.M. (2011). Numerical analysis of a rapid magnetic microfluidic mixer. Electrophoresis.

[27] Park S., Chuang H.-S., Kwon J.-S. (2021). Numerical study and taguchi optimization of fluid mixing by a microheater-modulated alternating current electrothermal flow in a y-shape microchannel. Sens. Actuators B Chem..

[28] Khakpour A., Ramiar A. (2020). Numerical investigation of the effect of electrode arrangement and geometry on electrothermal fluid flow pumping and mixing in microchannel. Chem. Eng. Process.-Process Intensif..

[29] Kunti G., Bhattacharya A., Chakraborty S. (2017). Analysis of micromixing of non-newtonian fluids driven by alternating current electrothermal flow. J. Non-Newton. Fluid Mech..

[30] Ng W.Y., Goh S., Lam Y.C., Yang C., Rodriguez I. (2009). Dc-biased ac-electroosmotic and ac-electrothermal flow mixing in microchannels. Lab Chip.

[31] Li Z., Kim S.J. (2017). Pulsatile micromixing using water-head-driven microfluidic oscillators. Chem. Eng. J..

[32] Rashidi S., Bafekr H., Valipour M.S., Esfahani J.A. (2018). A review on the application, simulation, and experiment of the electrokinetic mixers. Chem. Eng. Process.-Process Intensif..

[33] Zambrano H.A., Vasquez N., Wagemann E. (2016). Wall embedded electrodes to modify electroosmotic flow in silica nanoslits. Phys. Chem. Chem. Phys..

[34] Song H., Cai Z., Noh H.M., Bennett D.J. (2010). Chaotic mixing in microchannels via low frequency switching transverse electroosmotic flow generated on integrated microelectrodes. Lab Chip.

[35] Bag N., Bhattacharyya S. (2018). Electroosmotic flow of a non-newtonian fluid in a microchannel with heterogeneous surface potential. J. Non-Newton. Fluid Mech..

[36] Bhattacharyya S., Bera S. (2015). Combined electroosmosis-pressure driven flow and mixing in a microchannel with surface heterogeneity. Appl. Math. Model..

[37] Kateb M., Kolahdouz M., Fathipour M. (2018). Modulation of heterogeneous surface charge and flow pattern in electrically gated converging-diverging nanochannel. Int. Commun. Heat Mass Transf..

[38] Nayak A.K. (2014). Analysis of mixing for electroosmotic flow in micro/nano channels with heterogeneous surface potential. Int. J. Heat Mass Transf..

[39] Mahapatra B., Bandopadhyay A. (2020). Electroosmosis of a viscoelastic fluid over non-uniformly charged surfaces: Effect of fluid relaxation and retardation time. Phys. Fluids.

[40] Ahmed F., Kim K.Y. (2017). Parametric study of an electroosmotic micromixer with heterogeneous charged surface patches. Micromachines.

[41] Hwang H., Park J.K. (2011). Optoelectrofluidic platforms for chemistry and biology. Lab Chip.

[42] Baigl D. (2012). Photo-actuation of liquids for light-driven microfluidics: State of the art and perspectives. Lab Chip.

[43] Han D., Park J.K. (2016). Optoelectrofluidic enhanced immunoreaction based on optically-induced dynamic ac electroosmosis. Lab Chip.

[44] Pei-Yu C., Ohta A.T., Jamshidi A., Hsin-Yi H., Wu M.C. (2008). Light-actuated ac electroosmosis for nanoparticle manipulation. J. Microelectromech. Syst..

[45] Chiou P.Y., Ohta A.T., Wu M.C. (2005). Massively parallel manipulation of single cells and microparticles using optical images. Nature.

[46] Ding H.H., Zhong X.T., Liu B., Shi L.Y., Zhou T., Zhu Y.G. (2021). Mixing mechanism of a straight channel micromixer based on light-actuated oscillating electroosmosis in low-frequency sinusoidal ac electric field. Microfluid. Nanofluidics.

[47] Zhao Y., Hu S., Wang Q. (2013). Simulation and analysis of particle trajectory caused by the optical-induced dielectrophoresis force. Microfluid. Nanofluidics.

[48] Zhu X., Yin Z., Ni Z. (2011). Dynamics simulation of positioning and assembling multi-microparticles utilizing optoelectronic tweezers. Microfluid. Nanofluidics.

[49] Fu H., Liu X., Li S. (2017). Mixing indexes considering the combination of mean and dispersion information from intensity images for the performance estimation of micromixing. RSC Adv..

